# Honey bees and social wasps reach convergent architectural solutions to nest-building problems

**DOI:** 10.1371/journal.pbio.3002211

**Published:** 2023-07-27

**Authors:** Michael L. Smith, Kevin J. Loope, Bajaree Chuttong, Jana Dobelmann, James C. Makinson, Tatsuya Saga, Kirstin H. Petersen, Nils Napp

**Affiliations:** 1 Department of Collective Behaviour, Max Planck Institute of Animal Behavior, Konstanz, Germany; 2 Centre for the Advanced Study of Collective Behaviour, University of Konstanz, Konstanz, Germany; 3 Department of Biological Sciences, Auburn University, Auburn, Alabama, United States of America; 4 Department of Fish and Wildlife Conservation, Virginia Tech, Blacksburg, Virginia, United States of America; 5 Meliponini and Apini Research Laboratory, Department of Entomology and Plant Pathology, Faculty of Agriculture, Chiang Mai University, Chiang Mai, Thailand; 6 Institute of Evolutionary Ecology and Conservation Genomics, University of Ulm, Ulm, Germany; 7 Hawkesbury Institute for the Environment, Western Sydney University, Penrith, Australia; 8 Graduate School of Human Development and Environment, Kobe University, Kobe, Japan; 9 Department of Electrical and Computer Engineering, Cornell University, Ithaca, New York, United States of America; Centre National de la Recherche Scientifique, FRANCE

## Abstract

The hexagonal cells built by honey bees and social wasps are an example of adaptive architecture; hexagons minimize material use, while maximizing storage space and structural stability. Hexagon building evolved independently in the bees and wasps, but in some species of both groups, the hexagonal cells are size dimorphic—small worker cells and large reproductive cells—which forces the builders to join differently sized hexagons together. This inherent tiling problem creates a unique opportunity to investigate how similar architectural challenges are solved across independent evolutionary origins. We investigated how 5 honey bee and 5 wasp species solved this problem by extracting per-cell metrics from 22,745 cells. Here, we show that all species used the same building techniques: intermediate-sized cells and pairs of non-hexagonal cells, which increase in frequency with increasing size dimorphism. We then derive a simple geometric model that explains and predicts the observed pairing of non-hexagonal cells and their rate of occurrence. Our results show that despite different building materials, comb configurations, and 179 million years of independent evolution, honey bees and social wasps have converged on the same solutions for the same architectural problems, thereby revealing fundamental building properties and evolutionary convergence in construction behavior.

## Abstract 2

Honey bees and social wasps independently evolved hexagonal cells in their nests, but in some species, cell size dimorphism creates an inherent tiling problem. This study shows that despite independent evolutionary origins, building materials, and comb configurations, all species solve this architectural problem using the same solution.

Social insect nests are remarkable feats of construction; their structure is critical to colony success, but the scale is beyond the scope of any individual builder [1–3]. Nest architecture varies across species, reflecting aspects of their natural history that lead to building challenges (e.g., building nests within a defined cavity, exposed to the elements, or in conjunction with nest features that provide defense [4–6]). Unique building conditions make it difficult to directly compare nest architecture across species, but there is a *shared* challenge for *all* species that build hexagonal cells that exhibit size dimorphism [4,5,7]. Workers in these colonies typically build small hexagonal cells for rearing workers, and large hexagonal cells for rearing reproductives, which creates an inherent tiling problem: how to join non-uniform hexagons within a single lattice. Comparing how different species join non-uniform hexagons into a single array provides a rare opportunity to reveal how similar architectural challenges are solved across independent evolutionary origins.

Honey bees and social wasps represent independent origins of both eusociality and comb building [8]. In both groups, workers build hexagonal cells, though honey bees (*Apis*) build a double-sided array out of wax, whereas wasps typically build a single-sided array out of paper [4,6,9,10]. Building materials, however, are not free—honey bees must consume honey to produce wax, and wasps must spend time collecting wood and water to produce pulp [4,10–12]. Consequently, workers are expected to be frugal in their use of building material, and regular hexagonal cells are in fact optimal in the absence of boundary conditions [13]. We would also expect them to have evolved near-optimal behaviors when presented with the challenge of transitioning between cell sizes. While non-hexagonal cells exist in the hexagonal arrays of both honey bees and social wasps [7,14–17], these irregular shapes are usually ignored in previous research because measuring them is notoriously tedious. However, it is exactly in those situations, where construction of cells requires a non-trivial trade-off between material and functionality, that we may assess their skills as builders.

Here, we investigate hexagonal lattices built by 5 species of honey bees and 5 species of social wasps, representing at least 4 transitions between hexagonal cell size monomorphism and dimorphism in lineages separated by 179 million years [8] (Fig 1). Workers build reproductive cells when colonies reach a critical colony size, time of year, or colony condition, but the 2 cell sizes are constructed within the same hexagonal lattice [18–23]. For each species, we collected nest images that included both worker and reproductive cells (male cells for *Apis*, queen cells for *Vespula*). Specialized hexagonal reproductive cells are not built haphazardly throughout the comb, but instead appear clustered, with a clear transition between worker and reproductive cells. After identifying worker-to-reproductive transitions, we used a custom software [7] to identify and extract per-cell metrics. Each image and its corresponding metrics were verified by hand, to confirm the accuracy of both hexagonal and non-hexagonal cells. Using this dataset, which includes 22,745 cells extracted from 115 images, we investigated how each species solves the problem of tiling non-uniform hexagons into a single lattice.

**Fig 1:**
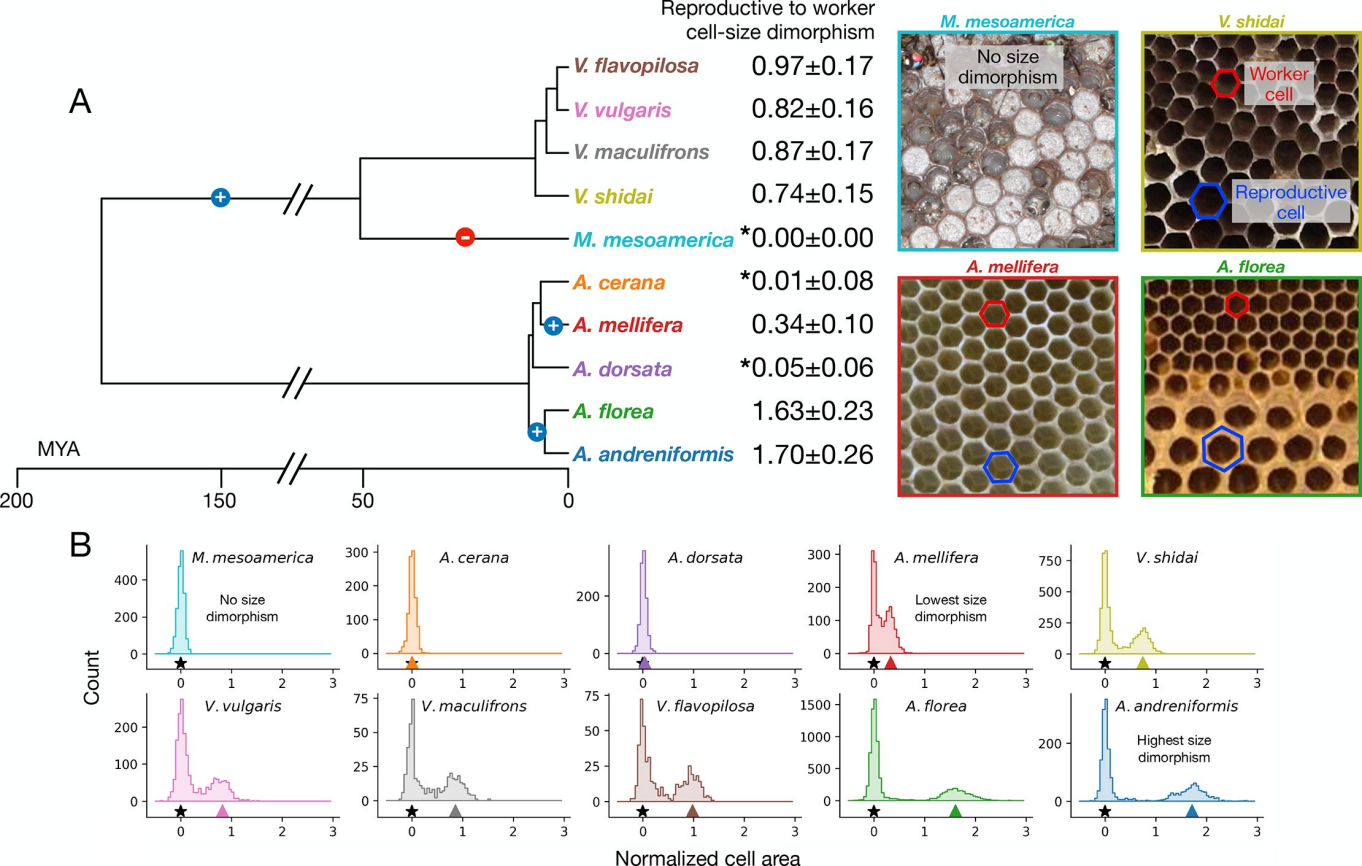
Size dimorphism in the hexagonal cells of honey bees and social wasps. (**A**) Phylogeny of the species investigated, with measurements of size dimorphism, and examples of comb images at right. Blue + indicates an independent origin of size dimorphism. Caste dimorphism evolved early in the eusocial wasps; thus, we assume a loss of caste dimorphism (red −) in the *M*. *mesoamerica* lineage. In *Apis*, 2 origins of dimorphism are parsimonious. (**B**) Histograms of cell area normalized by median worker cell area. Note that in species with a bimodal distribution (*A*. *mellifera* onward), the larger cells are used for rearing reproductives (black star at 0; colored triangle marks the median cell area for reproductive cells). Underlying data can be found at http://dx.doi.org/10.35099/aurora-605.

## Hexagonal size dimorphism across species

We first measured size dimorphism in each species by comparing the relative size of worker cells and reproductive cells (Fig 1). Dimorphism scores, i.e., additional area of reproductive cells relative to worker cells (D = [Reproductive cell area] / [Worker cell area] − 1) ranged from D = 0.0 (*Metapolybia mesoamerica*, no size dimorphism, based on lack of morphological queen caste [24]) to D = 1.70 (*Apis andreniformis*, highest size dimorphism; reproductive cells are 2.70 times larger than the median worker cell; Fig 1). For 2 species, *Apis cerana* and *Apis dorsata*, size dimorphism is essentially nonexistent (dimorphism scores of D = 0.01 and D = 0.05, respectively). Reproductive cells in these species were only identifiable by the capping on the cell (perforated in *A*. *cerana*; domed in *A*. *dorsata*) or by removing the cell capping to reveal the developing brood within (*Apis* males have enlarged compound eyes [5]). Given the phylogeny in Fig 1, and that *Apis mellifera* does have size dimorphism (D = 0.34), these results suggest that 2 origins of hexagonal cell dimorphism in *Apis* is the most parsimonious scenario. More complicated alternatives include (a) loss of size dimorphism in the shared ancestor of *A*. *mellifera*, *A*. *cerana*, and *A*. *dorsata* and a gain in *A*. *mellifera*; or (b) 2 independent losses of size dimorphism in *A*. *cerana* and *A*. *dorsata*. In social wasps, reproductive worker dimorphism is ancestral [25], and the monomorphism of *M*. *mesoamerica* is therefore due to a loss of dimorphism. These results allowed us to organize species according to the magnitude of their cell dimorphism, with the prediction that higher size dimorphism results in a more difficult tiling problem than species with low size dimorphism.

In species without size dimorphism, reproductives are reared alongside workers and distributed throughout the comb [5]. As size dimorphism increases, however, the architectural advantage of clustering specialized cells increases, as this reduces wasted space. Once reproductives are reared in specialized areas, workers can also adjust their rearing conditions (e.g., temperature profile and developmental time [26–28]). Therefore, the constraints imposed by size dimorphism not only alter the overall organization of the nest but also facilitate developmental divergence in the reproductives, which presumably can lead to further dimorphism.

### Different species use the same architectural solutions

**Fig 2:**
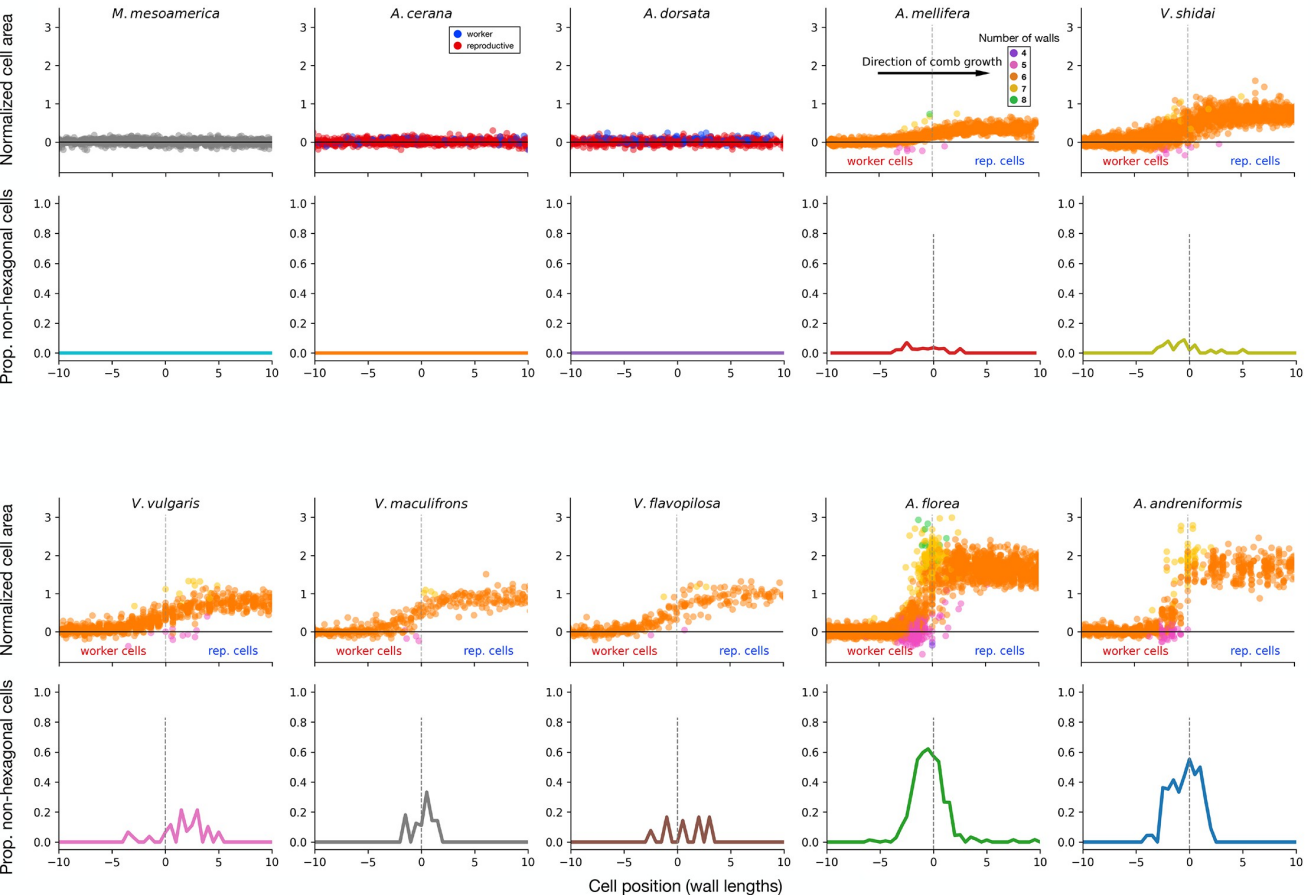
Individual cells across the worker-to-reproductive cell transition. Species are ordered left to right according to increasing size dimorphism (rows 1 and 3: normalized cell areas; rows 2 and 4: proportion of non-hexagonal cells). In *M*. *mesoamerica*, data points shown in grey, as worker and reproductive cells are indistinguishable. In *A*. *cerana* and *A*. *dorsata*, reproductive cells (blue) are built intermingled with worker cells (red). In species with size dimorphism (*A*. *mellifera* onward), reproductive cells are clumped. Worker cells on the left, reproductive cells on the right, with the vertical dotted line showing the transition point. Color denotes number of cell walls. Cell position (x-axis) is plotted as wall lengths for each species. In species without size dimorphism (no vertical dotted line), cells are centered relative to the image. Underlying data can be found at http://dx.doi.org/10.35099/aurora-605.

In species with size dimorphism, clusters of reproductive cells display a clear transition from their worker-sized counterparts. To reveal what changes in building behavior occurred across this transition, we aligned each cell relative to its distance from the transition point (the point at which cells change from worker to reproductive; vertical dotted lines in Fig 2). Strikingly, all species exhibited a combination of 2 construction behaviors to form the transition: Workers built intermediate-sized hexagonal cells to adapt to the larger-sized reproductive cells and incorporated non-hexagonal cells at the transition point (Fig 2). These non-hexagonal cells included 4-, 5-, 7-, and 8-sided cells, though 5- and 7-sided cells were the most common, together accounting for over 97.17% of all non-hexagonal cells (total non-hexagonal cells: 566; 4-sided: 2, 0.35%; 5-sided: 291, 51.41%; 7-sided: 259, 45.76%, 8-sided: 14, 2.47%).

**Fig 3:**
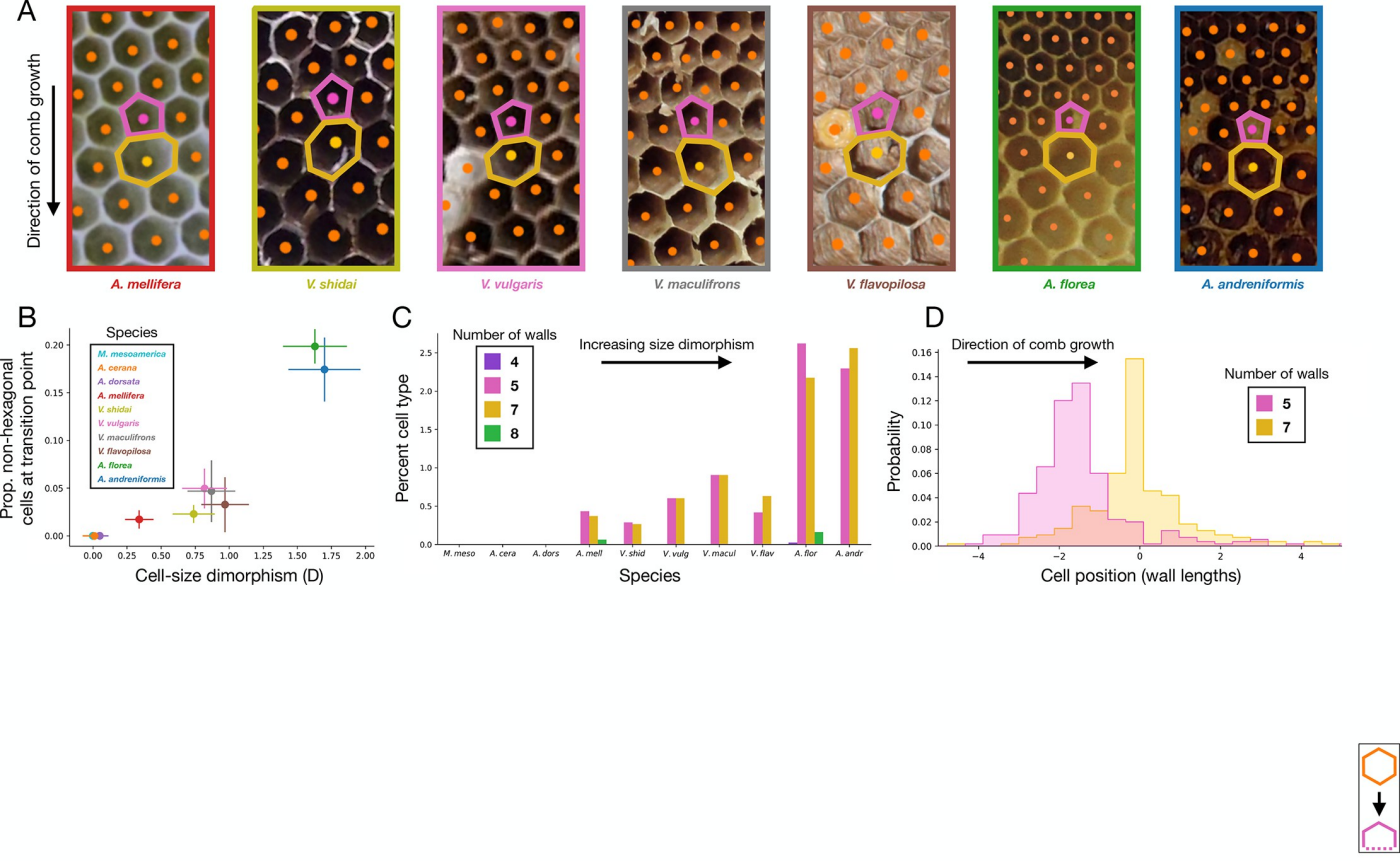
Workers build non-hexagonal cells. (**A**) Examples of 5–7 pairs in the species with size dimorphism, oriented with the direction of comb growth facing downwards. Note how the lower wall of the 5-sided cell has been truncated. (**B**) As size dimorphism increases, so too does the proportion of non-hexagonal cells at the transition point. Error bars show 95% CI. (**C**) The most common non-hexagonal cells are 5- and 7-sided, which are built in pairs. (**D**) Position of 5- and 7-sided cells shows that 5-sided cells are built first, followed by a 7-sided cell. Underlying data can be found at http://dx.doi.org/10.35099/aurora-605.

Non-hexagonal cells were completely absent in the 3 species with negligible size dimorphism (*M*. *mesoamerica*, *A*. *cerana*, *A*. *dorsata*). As the magnitude of size dimorphism increased, however, so too did the proportion of non-hexagonal cells at the transition point (Figs 2 and 3). This relationship held for all species (Fig 3) and was significant even when controlling for non-independence due to phylogeny (PGLS: ML lambda = 0.06; F_1, 8_ = 45.5; *p* < 0.001; adjusted R^2^ = 0.83). The pattern is also present when considering only the 7 dimorphic species, indicating a clear positive relationship not solely driven by the contrasts between the presence and absence of dimorphism (PGLS: ML lambda = 0.0; F_1, 5_ = 14.8; *p* = 0.01; adjusted R^2^ = 0.70). Therefore, despite multiple independent evolutions of comb building, and size dimorphism, their architectural solutions all fall upon the same continuum of non-uniform cell construction.

### Consistent configurations of non-hexagonal cells

The most common non-hexagonal cells were 5- and 7-sided. Curiously, the proportions of these cells were matched within each species (Fig 3). Pairs of 5- and 7-sided cells are known to exist in *A*. *mellifera* and are even found in graphene, an abiotic hexagonal lattice, which suggests a basic geometric reason for this pairing [7,15,17,29,30]. Here, we show that paired 5- and 7-sided cells are an architectural feature shared across all species with size dimorphism.

Workers typically build worker-sized cells before they transition to reproductive-sized cells, which allowed us to orient each array of cells according to the order in which each cell was built. Viewing cells in the direction of comb growth shows that when inserting non-hexagonal cells, workers build a 5-sided cell first, followed by a 7-sided cell (Fig 3). The 5-sided cell is formed by truncating the lower vertex; replacing 2 walls and a vertex with a single wall. This wall is the first to form the 7-sided cell. This truncation–expansion is also reflected in cell areas: 5-sided cells are smaller, and 7-sided cells are larger, as compared to hexagonal cells (Figs 2 and S1). Indeed, 7-sided cells are larger even than reproductive cells, though the hexagonal lattice stabilizes at reproductive-sized cells (Fig 2).

### Modeling predicts scale and type of architectural solution

**Fig 4:**
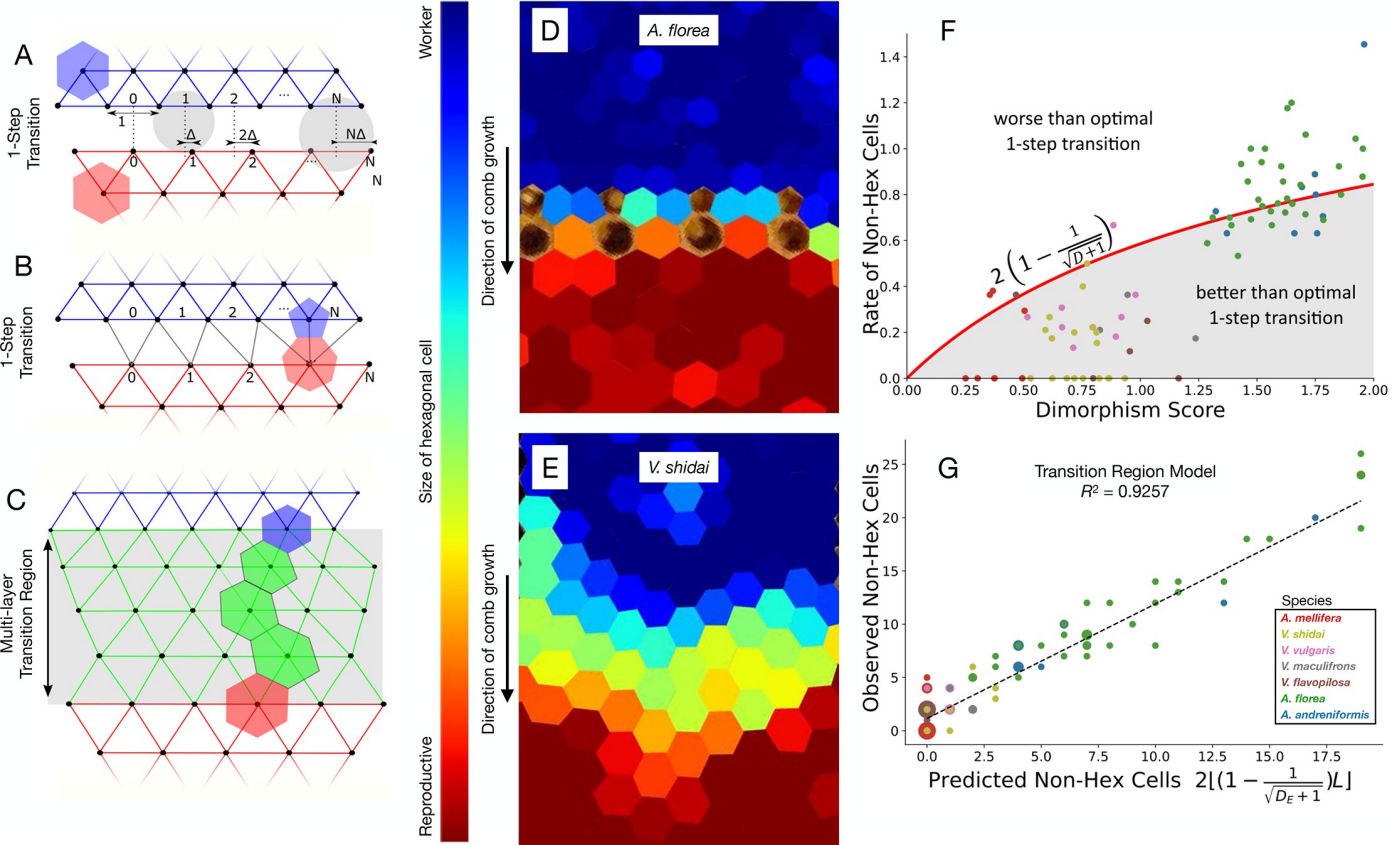
Modeling different merge strategies. (**A**-**C**) Connection pattern for transition between worker and reproductive cells, where the center of each additional reproductive cell is offset by Δ, the mismatch between worker and reproductive cells. (**A**) Lateral displacement of cell centers increasing left to right. The gray disks show the circumscribing circles for center triplets that would be connected in a regular lattice. Note that the disk on the right violates the Delaunay condition (disk contains 4, not 3, cell centers). (**B**) This lateral displacement introduces a 5–7 pair. (**C**) A transition region introduces intermediate-sized hexagonal cells, but no non-hexagonal cells (3 transition layers, in green). (**D**) Observed transition in *A*. *florea* showing a sudden transition between worker and reproductive cells, which includes 5–7 pairs (unshaded cells). (**E**) Observed transition in *V*. *shidai* showing a transition that uses intermediate-sized hexagonal cells, and no 5–7 pairs. (**F**) Observed rate of non-hexagonal cells per worker cell as a function of the observed size dimorphism (*n =* 115 images). The red line is the predicted rate based on a one-step worker-to-reproductive transition for an average transition length (strategy shown in **A** and **B**). The shaded area corresponds to transitions that outperform the best one-step transition in terms of non-hexagonal cells. Species with high size dimorphism do not build transition layers (*A*. *andreniformis*, *A*. *florea*; **D**), whereas species with moderate-sized dimorphism produce fewer non-hexagonal cells (“better than optimal” shaded region), by employing intermediate-sized cells across the transition region (strategy shown in **C**, **E**). (**G**) Regression of predicted non-hexagonal cells based on the following: size dimorphism, observed merge length, and depth of the transition region, versus the observed non-hexagonal cells, for each image. Marker size corresponds to the number of data points that fall on the same coordinate. In the model prediction (x-axis), each 5–7 pair produces 2 non-hexagonal cells. The observed non-hexagonal cells (y-axis) counts all non-hexagonal cells, including 4-sided, 8-sided, and unpaired cells. Underlying data can be found at http://dx.doi.org/10.35099/aurora-605.

We present a model that explains the particular pairing of 5- and 7-sided cells, the expected rate of such pairs based on the amount of size dimorphism, and the presence of intermediate-sized cells (Fig 4). Rather than focusing on a mechanistic model of how the animals build their nest, we start from 3 observations: (1) patches of worker and reproductive cells tend to be regular hexagonal and defect-free when far from the transition area; (2) the 2 patches of hexagonal cells generally have the same cell orientation; and (3) the transitions happen along a straight line. Given these observations, we then reasoned how non-hexagonal cells would arise if the builders acted optimally when transitioning between patches of worker and reproductive cells. The mathematical argument is based on studies of cells in *A*. *mellifera*, where the number of walls for each cell depends on the position of optimally spaced cell centers [7,30]. These cells are well represented by the polygons in a Voronoi partition, and in the following, we leverage the fact that in this construction the neighborhood graph of cell centers is a Delaunay triangulation, i.e., the circumscribing circle of each triangle in the graph contains no other centers (see Methods). Fig 4A shows an example of how cell centers from worker and reproductive cells can be combined while upholding (left gray disk) or violating (right gray disk) the Delaunay triangulation.

When there is size dimorphism, the mismatch of cell sizes on either side of the transition makes regular connection patterns impossible (e.g., there are 7 worker cells and 6 reproductive cells along the transition line in Fig 4A). We can quantify this effect as follows. First, assume cells on either side of the transition are aligned as they should be in a regular connection pattern (Fig 4A “0”) and then count the number of worker cell neighbors moving laterally away from this point ([1, 2, … N]). Each pair of worker cell neighbors and one reproductive cell should form one triangle of the triangulation. With each step the mismatch, $\Delta =\sqrt{D+1}-1$, between the cell centers accumulates, and once this mismatch is larger than half a reproductive cell (Fig 4A “N”), the circumscribing circle would encompass both the current and the previous reproductive cell center. Thus, to maintain the Delaunay condition, a worker cell essentially loses an edge in the graph and becomes 5-sided, whereas the previous reproductive cell gains an edge and becomes 7-sided (Fig 4B).

Following this line of reasoning results in an expected rate of 5-7 pairs. We show that a regular hexagonal connection pattern cannot be sustained for more than $\frac{\sqrt{D+1}}{\sqrt{D+1}-1}$ worker cell lengths before a 5-7-sided pair must be incorporated, with the 5-sided cell on the worker side of the transition and the 7-sided cell on the reproductive side (see Methods). This means that for a given one-step transition, we would expect to see $\left\lfloor (1-\frac{1}{\sqrt{D+1}})L\right\rfloor$ 5-7 pairs, where *L* is the length of the transition measured in number of worker cells lengths. When size dimorphism is low, workers can avoid building any non-hexagonal cells if the number of reproductive cells in the comb is small (i.e., the number of fractional predicted pairs can be rounded down, because the accumulation of mismatch, Δ, has not yet forced a 5-7 pairing; Fig 4A and 4B).

The observed data also show that some species use a second, complementary strategy to solve the transition problem: the incorporation of layers of intermediate-sized hexagonal cells to minimize the occurrence of non-hexagonal cells (Fig 4C, 4D and 4E). The geometrical argument also works for this strategy, basically treating the intermediate layers as a series of consecutive one-step low-dimorphism transitions.

In high-dimorphism species, e.g., *A florea* and *A*. *andreniformis*, we do not observe layers of intermediate-sized cells as this strategy would require many layers of cells with areas that are far outside the distributions of either worker or reproductive cells and therefore are presumably unsuitable for rearing brood [31]. We only observe intermediate-sized cell transitions in species with moderate dimorphism; these species incorporate 5-7 pairs at a lower rate because transitional layers essentially decrease the dimorphism score that forces 5-7 pairs. The fact that species with moderate dimorphism outperform the optimal number of 5-7 pairs in a one-step transition can be seen in Fig 4F, where the majority of data points for moderate dimorphism species fall in the “better than optimal” shaded region. We estimate the number of transition layers by fitting a gradual 4-parameter size transition model for each comb image. This produces the transition area, which is used to fit optimal transition layers—optimal in the sense that the size difference between consecutive layers is as big as possible without introducing 5-7 pairs. The “effective” dimorphism score, *D*_*e*_, now accounts for the remaining size difference that was not accommodated for by those transition layers (Fig 4G, Methods).

Although the geometric argument makes simplifying assumptions, the observed data fit the model well (Fig 4G). It predicts that non-hexagonal cells should occur as 5-7 pairs; of the observed 5- and 7-sided cells, 87.8% are in pairs as predicted (85.2% of all non-hexagonal cells). The model also recovers the observed positive relationship between the dimorphism score and the proportion of non-hexagonal cells. The sudden transitions produced by the high-dimorphism species, *A*. *andreniformis* and *A*. *florea* (D > 1.50), do not include many intermediate-sized hexagonal cells (Figs 1 and 2), and the observed rate of 5-7-sided pairs matches the one-step prediction (Figs 3 and 4). Species with no/low dimorphism (D < 0.10) do not produce any non-hexagonal cells, and species with moderate dimorphism (0.10 ≤ D ≤ 1.50) use layers of intermediate-sized hexagonal cells, as indicated by the cell size distribution and gradual transition (Figs 1 and 2), modeled in Fig 4G. In addition to being derived from simple geometric assumptions, using our model to predict the observed number of non-hexagonal cells across all images and species produces a better correlation (R^2^ = 0.93) than data-driven models that regress on the size dimorphism alone (Figs 4G and S2).

## Conclusions

Investigating how architectural challenges are overcome in different biological systems provides a rare opportunity to directly compare the outcomes of collective building. While our work has focused on cell configurations in completed combs, a finished product of collective behavior, future work could address the mechanistic and behavioral processes of non-hexagonal cell construction. For example, are 5-7 pairs built in their entirety during the transition to reproductive cells, or do workers rearrange walls to achieve this configuration? In *Apis*, the comb building area is obscured by a thick layer of bees, but X-ray tomography can be used to visualize comb growth beneath the cluster [32]. Social wasps, however, do not cluster as tightly, and so could be a preferred system, especially in species that exhibit high rates of size dimorphism, and, therefore, 5-7 pairs are more frequent. It is possible that the building materials themselves, wax versus paper, influence the ways in which builders must adapt their building behavior to accommodate non-hexagonal configurations within their latticework.

Here, we show that despite different building materials, comb configurations, costs, and evolutionary origins, the honey bees and social wasps all solve the architectural problem of size dimorphism with the same scalable solutions; intermediate-sized cells, and non-hexagonal cells, arranged in configurations that can be predicted by assuming near-optimal building behavior in simplified geometries. Unlike species-specific rules [33], these results suggest that the evolved behavior of all species have converged upon the same solution for a fundamental geometrical reason. Models of distributed construction tend to focus on higher-level structures in idealized conditions [34]. By focusing on building responses to biologically relevant cell configurations, where “perfectly” symmetrical hexagons cannot be built, we show evidence for convergent evolution in architectural outcomes across independent origins of comb construction.

## Methods

### Image collection

Each nest image had to have individually discernable cells, including cell walls and vertices. For managed species of *Apis*, combs had to be built without embossed wax or plastic foundation, as this would alter their building behavior. For the social wasps, nests had to be free of wooden sticks or other materials that would alter cell shapes. Most combs in *Vespula* nests consist entirely of worker or queen cells; for our analysis, we only selected images of comb that include regions of both cell types.

Images of *A*. *andreniformis* and *A*. *cerana* nests were taken in the Mae Rim district of Chiang Mai, and Mae Fah Leuang University (Thailand). Images of *A*. *dorsata* nests were taken in the Mae Rim district of Chiang Mai (Thailand). Images of *A*. *florea* nests were taken in the Mae Rim district of Chiang Mai, Mae Fah Leuang University, and the Ratchaburi campus of King Mongkut University (Thailand). Images of *A*. *mellifera* nests were taken at the Liddell Field Station of Cornell University, in Ithaca, NY (USA). Images of *M*. *mesoamerica* nests were taken at La Selva Biological Station (Costa Rica; permit number 018-2010-SINAC). Images of *V*. *flavopilosa* nests were taken in Ithaca NY (USA). Images of *V*. *maculifrons* nests were taken in Georgia and North Carolina (USA). Images of *V*. *shidai* nests were taken at Gifu and Nagano (Japan). Images of *V*. *vulgaris* nests were taken at the Nelson Lakes National Park (New Zealand), and the Katholieke Universiteit Leuven (Belgium). We further supplemented our dataset by performing web search for nest images of common species (e.g., *A*. *florea*) and by contacting individual researchers.

For each species, we collected the following number of images and cells: *A*. *andreniformis*, 9 images, 1,875 cells; *A*. *cerana*, 12 images, 1,030 cells; *A*. *dorsata*, 9 images, 1,085 cells; *A*. *florea*, 33 images, 8,046 cells; *A*. *mellifera*, 8 images, 1,619 cells; *M*. *mesoamerica*, 8 images, 1,834 cells; *V*. *flavopilosa*, 5 images, 477 cells; *V*. *maculifrons*, 3 images, 442 cells; *V*. *shidai*, 19 images, 4,511 cells; *V*. *vulgaris*, 9 images, 1,827 cells.

### Semi-automated image analysis of comb cells

To extract per-cell metrics, we used a custom GUI to identify cell centers, vertices, and walls (see details in [7]). The original software was intended for images taken under controlled lighting conditions, but the images used in this study had more heterogeneous conditions. Therefore, each cell in each image was verified, by hand, to confirm that the position of the cell center, vertices, and walls accurately matched the cells in the image. This semi-automated method allowed us to include cells from images that would otherwise not have been feasible (e.g., nest images that still had the occasional adult worker atop the comb, as long as the cell was still visible underneath). For each image, we identified the location of worker cells, reproductive cells, and the transition between the two. In some instances, the cells across the full transition region could not be identified (e.g., if multiple consecutive cells were damaged, or a foreign object was embedded in the nest).

### Statistical analyses

Statistical analyses were performed in Python (ver. 3.8.9), and R (ver. 4.0.2). Scale bars were not present in most images, so we instead normalized our measurements relative to worker-sized cells (i.e., cell area 0 = median worker cell area; cell area 1.5 = cell is 1.5 times larger than the median worker cell). To keep plots consistent across species, data are plotted in units of worker-wall lengths (e.g., distance from transition point, in units of worker-wall lengths). This made comparisons across species feasible, as the units of measurement are scaled to each species, and independent of image quality.

### Phylogeny and comparative methods

First, we created a phylogenetic tree of our species using divergence dates (in millions of years ago) from key nodes in [8] (split between Vespidae and Apidae; split between Vespinae and Polistinae) and [35] (splits between species of *Apis*). We could not find a time-calibrated tree containing our species of *Vespula*. Instead, we used the topology described in [36] with *V*. *flaviceps* as a proxy for *V*. *shidai* (these are sister species [37]), with node ages set to one less than the number of descending species [38]. We then linearly scaled the branch lengths of the *Vespula* clade so that the divergence time between *V*. *shidai* and the remaining species was 8 mya, as this occurred prior to the divergence between *V*. *germanica* and *V*. *vulgaris* [36], estimated at 6 mya [39].

To control for non-independence of species-level data due to shared evolutionary history, we used phylogenetic least squares regression (PGLS) to determine if the degree of cell size dimorphism predicted the percentage of cells that are non-hexagonal. We used the pgls function in the package *caper* to simultaneously estimate the maximum likelihood value for the branch-length scaling parameter *lambda*, which finds the best degree of phylogenetic scaling that minimizes the phylogenetic correlation of the residuals of the regression, while also fitting the regression between dimorphism and proportion of non-hexagonal cells [40]. We also created a PGLS model with only the species with substantial size dimorphism (excluding *M*. *mesoamerica*, *A*. *cerana*, and *A*. *dorsata*) to see if we could find evidence for an effect of the degree of dimorphism on the proportion of non-hexagonal cells.

### Geometric model for non-uniform hexagonal lattice structure

This material covers details related to the geometrical argument for how different-sized hexagons can be joined in a single lattice structure, as related to comb built by honey bees and social wasps.

#### 1. Units

In species with sexual size dimorphism, reproductive cells are larger than worker cells, by a factor *α*, so that the regular hexagonal pattern of a comb is disturbed when the colony transitions to building reproductive cells. Here, *α* refers to the linear scale difference between worker and reproductive cells. Note that some figures report the area increase *D*, as appropriate in the context of the discussion (Figs 1 and 2). To convert between the two: *D* = *α*^2^−1 and $\alpha =\sqrt{D+1}$. Throughout the model description, we further refer to *L* as the length of the transition line between the worker and reproductive cell patches, measured in wall-to-wall worker cell widths.

#### 2. Assumptions and model

Our goal is to derive a simple relation that can explain the observed cell geometries in the transition regions of naturally constructed comb, i.e., how many non-hexagonal cells to expect along a transition line between worker and reproductive cells (Fig 4A–4C). This relation is then used to predict the number of expected non-hexagonal cells for all species, whether or not they use layers of intermediate sized cells.

When we assume that the transition happens suddenly, from one worker cell to the next reproductive cell, we can derive the relation $2(\frac{\alpha -1}{\alpha })$ as the number of non-hexagonal cells we would expect per worker cell along a transition line. Furthermore, we would expect 5-7-sided cell pairs where the 5-sided cell is a deformed worker cell and the 7-sided cell is a deformed reproductive cell. This agrees with current and previous observations [7,30].

To start, we assume a patch of regularly spaced worker cells and a (not yet built) patch of reproductive cells. We then examine how these 2 patches could be connected over a sudden transition, and when the difference in cell size requires non-hexagonal cells. Instead of directly analyzing the placement of cell walls, we look at the dual representation of a comb in terms of its cell centers (marked with black points in Fig 4A–4C). To relate the cell center representation to cells, we use the centers to generate a Voronoi partition to decompose the plane into polygonal areas, i.e., polygons that have the properties that all the points inside a polygon are closest to the cell center that is inside the partition, and all the edges are equidistant from 2 cell centers. Prior studies on irregular nest construction found that the Voronoi partition generated by cell centers is in good agreement to the observed cell walls [30]. To derive a bound on the number of necessary non-hexagonal cells, we use the fact that the graph generated by connecting centers whose Voronoi partition polygons share an edge is a Delaunay triangulation, i.e., a graph where any circumscribing circle of a triangle contains no other vertices. This equivalence is helpful since the connection topology of cell centers is easier to analyze and we can derive bounds based on enforcing the Delaunay condition.

Next, we analyze the graph of cell centers for both patches and assume a partial Delaunay triangulation where all edges that are purely between worker cell centers and reproductive cell centers are already added. Now we can add the remaining edges and analyze the connection topology as allowed by the Delaunay condition to compute the fraction of non-hexagonal cells along the transition line (Fig 4A–4C).

From the perspective of a worker cell edge that faces the reproductive cell patch, we need to choose a cell center from the reproductive cells that completes the triangle. Since we already know 2 points of the triangle, we know that the center of the circumscribing circle needs to be located on the bisecting normal of that edge (and if the reproductive cells were the same size, the center would be *on* that bisecting line). By symmetry around the bisecting line, the reproductive cell center that is chosen cannot be further away than $\frac{\alpha }{2}$ from the bisecting line since the circumscribing circle would otherwise contain another vertex and violate the Delaunay condition.

To compute the number of hexagonal cells that can be connected before non-hexagonal cells need to be introduced, assume that we connect one cell and that the reproductive cell is perfectly coincident with the bisecting line (marked by a “0” in Fig 4A and 4B). Therefore, the next reproductive cell center is Δ = (*α*−1) away from the bisecting line, and the next cell is 2(*α* − 1), etc. After *N* worker cell edges, the corresponding reproductive cell center is *N*(*α*−1) from the bisecting line. When *α >* 1, this will eventually violate the Delaunay condition and $N(\alpha -1)>\frac{\alpha }{2}$ such that the triangulation should instead connect to the *N*−1st reproductive cell center (gray shaded disk on the right in Fig 4A). This dislocation from the regular connection pattern removes one edge from a worker cell and adds one edge to a reproductive cell resulting in a 5-7-sided pair of cells (i.e., 2 non-hexagonal cells per dislocation; red and blue shaded cells in Fig 4B). The same reasoning applies whether we consider cells to the right or the left of the 0^th^ reproductive cell center, so that for $N>2\frac{\alpha }{2(\alpha -1)}=\frac{\alpha }{\alpha -1}$, there must be a dislocation resulting in a 5-7-sided cell pair. In other words, on average, we would expect $\frac{1}{L}=\frac{\alpha -1}{\alpha }$ dislocations per worker cell along the transition line, and twice as many non-hexagonal cells.

#### 3. Intermediate-sized cell transitions and mixed strategies

Due to the discretization of 5-7-sided cell pairs, it is possible to achieve transitions along a length of $L_{max}=\frac{\alpha }{\alpha -1}$ without having to introduce any non-hexagonal cells. For example, for low-dimorphism species, where α is close to one, *L*_max_ is large, and we would not expect to need non-hexagonal cells. Conversely, given a length *L*, we can compute the largest size difference than can be accomplished without having to introduce dislocations (and associated 5-7 pairs). For a particular *L*, the maximum *α*_*L*_ that can be transitioned to without introducing any dislocations is $\frac{L}{L-1}$, and we can use this expression to examine strategies that include building intermediate sized cells.

Let *T* denote the number of “transition layers”, i.e., hexagonal cells of intermediate size that accomplish the worker-reproductive transition without introducing non-hexagonal cells. Given an *α* associated with a given size dimorphism and an *α*_*L*_ associated with a particular transition, then a dislocation-free transition could be accomplished with *T* successive steps such that *α*_*L*_^*T*^ = *α*. The multiplicative nature of this relation is due to the fact that in the first layer, all the cells have the size *α*_*L*_ and increasing to the second layer the largest cells that can be achieved without introducing a 5-7 pair should be *α*_*L*_ * *α*_*L*_, and *α*_*L*_ * *α*_*L*_ * *α*_*L*_ for the third layer, etc.

We explored several strategies for estimating the number of transition layers from the image data. Given that regular cells have size variation, deciding if a particular cell is a regular cell or a transition cell is somewhat arbitrary, and we found that different confidence level cutoffs produced different results. Instead, we used a simple transition model with 4 parameters (*A*_*worker*_, *A*_*rep*_, *O*_*worker*_, *O*_*rep*_) to fit the areas of hexagonal cells using least squares, where the variables *A* correspond to the areas of worker and reproductive calls and the variables *O* correspond to the offsets from a line fit to the transition. All cells that are further than *O*_*worker*_ away from the transition line in one direction are assumed to be of *A*_*worker*_; all cells that are further away than *O*_*rep*_ in the other direction are assumed to be of size *A*_*rep*_. The cells between the 2 offsets are linearly interpolated between the worker and reproductive cell sizes. The distance between the 2 offsets in this fit is the measured length of the transition and used to estimate *T*_*obs*_ While somewhat simple, this model has no additional tuning parameters and only assumes the overall geometry of the merge instead of trying to classify individual cells as being part of the regular comb or transition regions.

When measuring transition layers from image data, the distance between the 2 offsets seldom exactly fits multiples of *α*_*L*_. In that case, we interpolate the last (largest) layer to estimate the total number of transition layers *T*_*obs*_. Since the introduction of non-hexagonal cells will deform the adjacent hexagonal cells, resulting in intermediate sizes, we require that the transition region contain at least 50% hexagonal cells. If it does not, we set *T*_*obs*_ to zero. This is only an issue for high-dimorphism species where the one-step transition has a high rate of 5-7 pairs and where building intermediate sized cells is clearly not part of their strategy (but does occur because of the adjacent non-hexagonal cells). Setting *T*_*obs*_ to zero in these cases prevents those transitions from being treated like a mixed strategy, described next.

Using *T*_*obs*_, we can also model a mixed strategy where some intermediate sized cells are introduced during the transition, but where *T*_*obs*_ is too small to complete the transition without introducing 5-7 pairs. For such “incomplete” transition layers that have both intermediate sized cells and 5-7 pairs, we can compute an effective *α*_*E*_, i.e., the size difference left after using transitional layers to narrow the difference, *α*_*E*_ *α*_*L*_^*Tobs*^ = *α* or *α*_*E*_ = = $\frac{\alpha }{\alpha_{E}^{T_{obs}}}$. In this situation, we would expect a rate of 5-7 pairs of $\frac{\alpha_{E}-1}{\alpha_{E}}$ (Fig 4G).

We observe different relative preference of having non-hexagonal or intermediate-sized cells dependent on species. For species where the worker and reproductive sized cells are widely separated, such as *A*. *florea*, a pure intermediate cell size transition would produce many layers of cells that are outside the range of either worker or reproductive cell sizes, which is not observed (Fig 4D and 4F). For species with intermediate-sized dimorphism, we do observe transition layers and intermediate-sized cells (Fig 4E). Comparing the quality of the model with and without transition layers is shown in S2 Fig, including the transition layer and using *α*_*E*_ does a better job (R^2^ = 0.93 versus R^2^ = 0.86) of predicting the rate of non-hexagonal cells for the entire dataset.

### Comparison to alternative models

In the main paper, we presented the best-fit model out of 4. Here, we elaborate on the alternative models evaluated (S2 Fig). The first and second model predicts the rate of non-hexagonal cells if it is directly proportional to the observed dimorphism score, D, and the observed mismatch between cells Δ = (*α*−1), respectively. Both were included only for comparison; they take a simple data-driven approach and do not explain the particular rate or configuration of the non-hexagonal cells. The third model predicts the rate of non-hexagonal cells as a one-step transition, $\frac{\alpha -1}{\alpha }$, and the fourth model includes a mixed strategy that can incorporate intermediate sized cells if they are observed in the image, $\frac{\alpha_{E}-1}{\alpha_{E}}$. We see that the third (one-step transition) model predicts the particular configuration of 5-7-sided cells but predicts a higher rate of non-hexagonal cells for moderate dimorphism species. The fourth model includes observed transition layers and does a better job of predicting the occurrence rate of non-hexagonal cells in medium-dimorphism species that build intermediate-sized cells. Including both medium- and high-dimorphism species, the *R*^2^ fit score of 0.93 is better than all the other approaches, and the coefficient of proportionality is close to one, indicating that our derived model does a good job of directly predicting the observed number of non-hexagonal cells despite the simplifying assumptions. Given that our model assumes optimal behavior, it inherently underestimates the number of non-hexagonal cells indicated by a coefficient greater than 1.

## Supporting information

S1 Fig Histogram of normalized cell areas for 5-, 6-, and 7-sided cells, showing that 5-sided cells tend to be smaller, and 7-sided cells tend to be bigger, than hexagonal cells. Underlying data can be found at http://dx.doi.org/10.35099/aurora-605.
(PDF)

S2 Fig Comparing models for predicting the occurrence rate of non-hexagonal cells. The x-axis for each plot is the predicted number of cells based on the model-predicted rate and the observed length of the transition regions. The y-axis is the observed number of non-hexagonal cells. The dashed black line is the best-fit linear regression for the prediction and the observed number of non-hexagonal cells. Since the numbers are all integers, multiple data points can fall on the same coordinate, so the area of the marker is proportional to the number of data points (colors denote species). The models shown in (**A**, **B**) are data-driven, whereas the models in (**C**, **D**) explain both the rate and particular configuration of non-hexagonal cells. Underlying data can be found at http://dx.doi.org/10.35099/aurora-605.
(PDF)
