# Supplementary figures and images for: Honey bees and social wasps reach convergent architectural solutions to nest-building problems
Source: PLoS Biol. 2023 Jul 27;21(7):e3002211. doi: 10.1371/journal.pbio.3002211 (PMC10374112; doi:10.1371/journal.pbio.3002211)

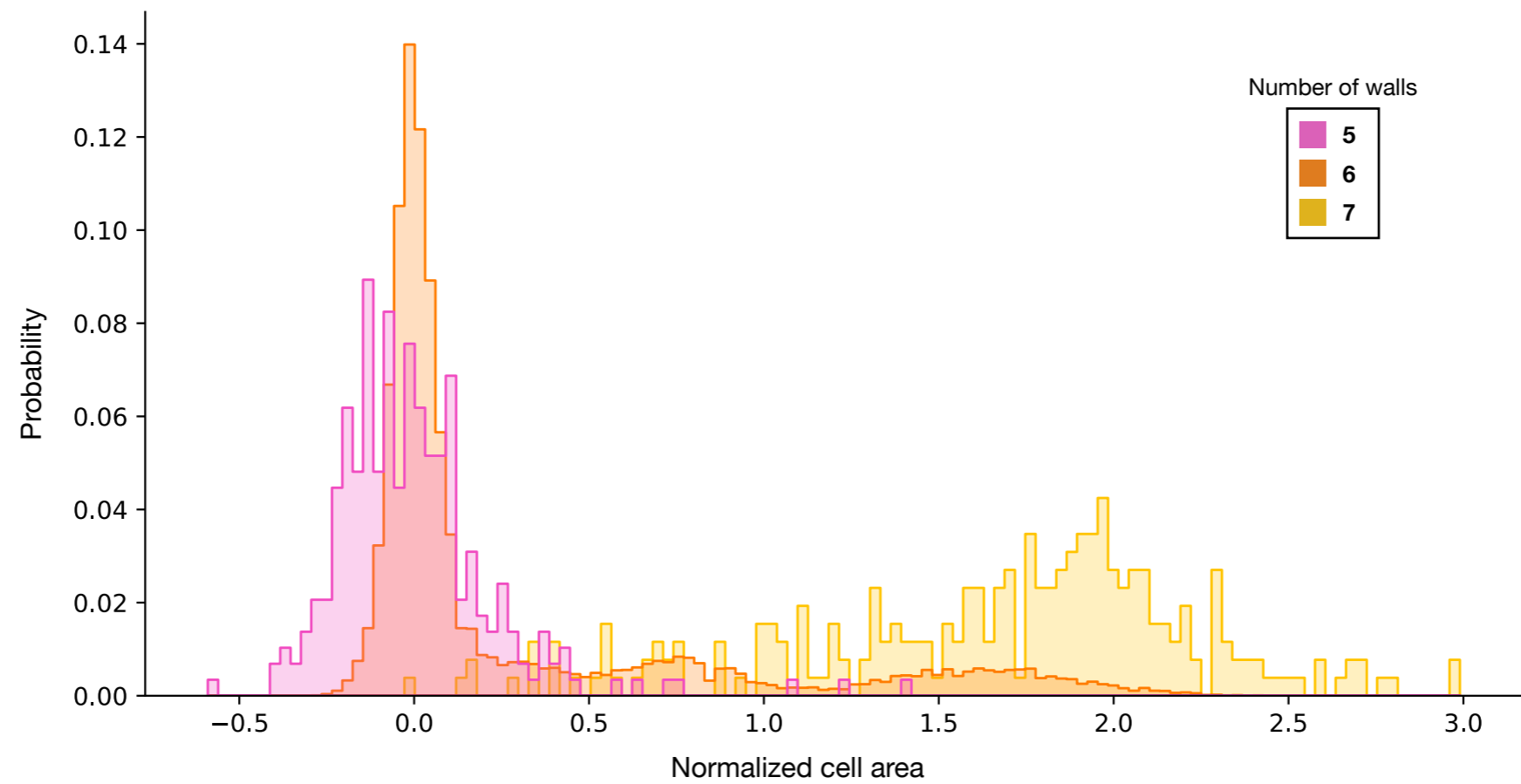

Supplement: S1 Fig — Underlying data can be found at http://dx.doi.org/10.35099/aurora-605. (PDF) [file pbio.3002211.s001.pdf]

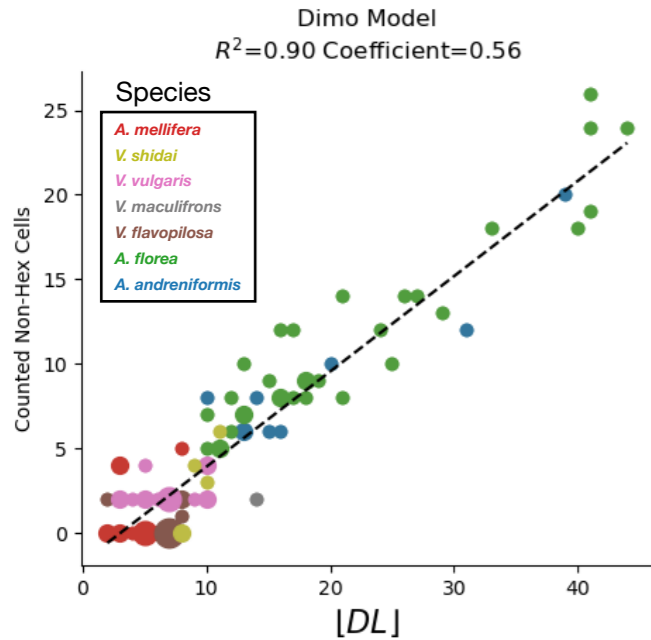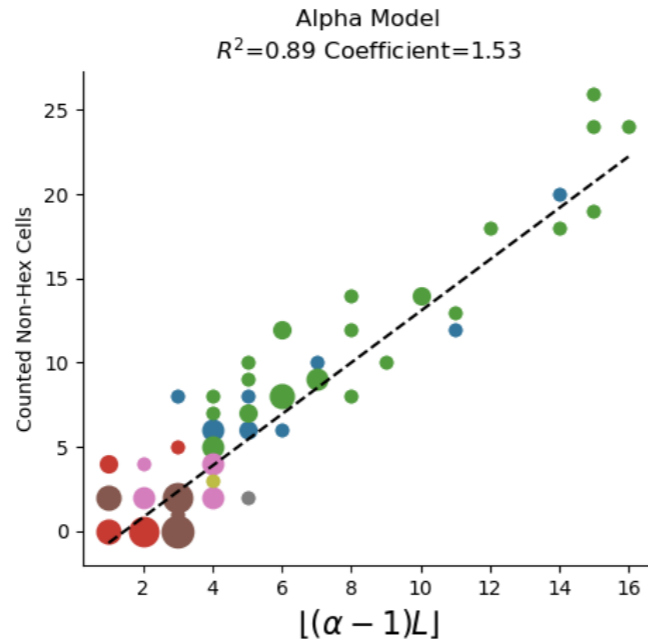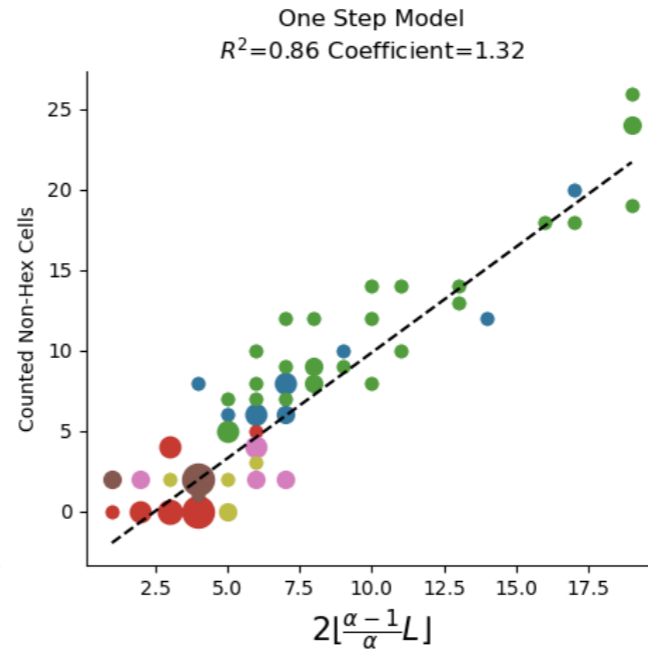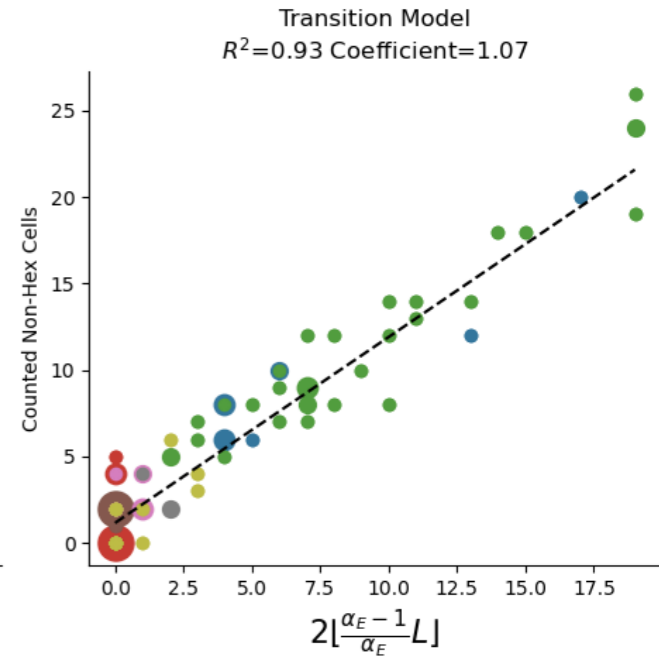

Supplement: S2 Fig — The x-axis for each plot is the predicted number of cells based on the model-predicted rate and the observed length of the transition regions. The y-axis is the observed number of non-hexagonal cells. The dashed black line is the best-fit linear regression for the prediction and the observed number of non-hexagonal cells. Since the numbers are all integers, multiple data points can fall on the same coordinate, so the area of the marker is proportional to the number of data points (colors denote species). The models shown in (A, B) are data-driven, whereas the models in (C, D) explain both the rate and particular configuration of non-hexagonal cells. Underlying data can be found at http://dx.doi.org/10.35099/aurora-605. (PDF) [file pbio.3002211.s002.pdf]
